# Analysis of the Specificity and Biochemical Characterization of Metalloproteases Isolated from *Eupenicillium javanicum* Using Fluorescence Resonance Energy Transfer Peptides

**DOI:** 10.3389/fmicb.2016.02141

**Published:** 2017-01-09

**Authors:** Youssef A. A. Hamin Neto, Lilian C. G. de Oliveira, Juliana R. de Oliveira, Maria A. Juliano, Luiz Juliano, Eliane C. Arantes, Hamilton Cabral

**Affiliations:** ^1^Department of Pharmaceutical Sciences, School of Pharmaceutical Sciences of Ribeirao Preto, University of São PauloRibeirão Preto, Brazil; ^2^Department of Biophysics, Paulista Medical School, Federal University of São PauloSão Paulo, Brazil; ^3^Department of Physics and Chemistry, School of Pharmaceutical Sciences of Ribeirao Preto, University of São PauloRibeirão Preto, Brazil

**Keywords:** biochemical characterization, fluorescence resonance energy transfer peptides, microbial enzyme, protease, solid-state fermentation

## Abstract

Enzymes have important features that may facilitate their application in industrial processes and have been used as alternatives to chemical catalysts. In particular, proteases can be isolated from microorganisms, which provide important sources of advantageous enzymes for industrial processes. For example, *Eupenicillium javanicum* is a filamentous fungus that has been shown to express industrially applicable enzymes and chemical components, such as antifungal compounds. The biotechnological potential of *E. javanicum* and proteases made us search a novel protease from this microorganism. The macromolecule was isolated, the main biochemical properties was evaluated, and the specificity of the protease subsites was determined. The protease was produced under solid-state bioprocess with wheat bran and isolated by two chromatography steps with yield of 27.5% and 12.4-fold purification. The molecular mass was estimated at 30 kDa. The N-terminal sequence of the first 20 amino acid residues was AVGAGYNASVALALEKALNN. The enzyme presented higher proteolytic activity at pH 6.0 and 60°C. The protease is stable at wide range of pH values and temperatures and in the presence of surfactants. The “primed” side of the catalytic site showed the highest catalytic efficiency of the enzyme isolated from *E. javanicum*. The S′_1_ subsite is responsible for catalyzing the protease reaction with substrates with tyrosine in P′_1_. These findings provide important insights into the biochemical characterization of a highly active protease from *E. javanicum* and may facilitate the development of industrial processes involving this protease.

## Introduction

Enzymes present some advantages when compared with chemical catalysts. These macromolecules are able to catalyze a variety of chemical reactions, industrial processes have been shifting to the application of enzymes rather than chemicals as catalysts (Sarrouh et al., 2012). In addition, enzymes have several advantages over chemical catalysts, such as mild reaction conditions, the lack of requirement for protection of substrate functional groups, long half-lives, high stereo selectivity, and the ability to be genetically modified to improve stability, substrate specificity, and specific activity (Adrio and Demain, 2014). The key properties of enzymes, including stable activity over a range of temperatures and pH values and broad catalytic specificity, can be exploited in various industries and processes, such as the food, laundry, detergent, leather, pharmaceutical, and silk industries; for recovery of silver from x-ray film; and for waste management (Nigam, 2013; Hmidet et al., 2015).

Proteases are hydrolytic enzymes that have been used in industrial processes for decades. Microorganisms under bioprocesses can act as sources of various proteases to improve the protease production and activity owing to their rapid growth and potential for genetic modification, thereby allowing scientists to enhance the features of proteases for applications in industrial processes (Souza et al., 2015).

The solid-state bioprocess present potential applications in the production of enzymes and other industrial products. It has been defined as bioprocess in the absence or near-absence of free water, with enough moisture to support the growth and metabolic activity of the microorganism. Other characteristic is the use of low-cost agro-industrial residues, it makes the solid-state fermentation attractive financially and sustainably (Thomas et al., 2013).

Among these microbial sources, the filamentous fungus *Eupenicillium javanicum* has been shown to produce different products with industrial interests, such as antifungal compounds (Nakadate et al., 2007, 2008), endoglucanase, β-glucosidase, pectinase, xylanase (Tao et al., 2011), and a protease stable to spray dryer process (Hamin Neto et al., 2014).

Therefore, in this study, we aimed to isolate a protease produced in *E. javanicum* during solid-state fermentation, evaluate the main biochemical properties of this protease, and determine the specificity of its subsites.

## Materials and Methods

### Isolation, Identification, and Maintenance of *E. javanicum*

The fungus *E. javanicum* was isolated from silage and belongs to a collection of microorganisms at the Enzyme Technology Laboratory under the supervision of Dr. Hamilton Cabral (Faculdade de Ciências Farmacêuticas de Ribeirão Preto, Universidade de São Paulo). The fungus could be maintained in Sabouraud medium at 4°C for up to 1 month.

### Inoculum Preparation

*Eupenicillium javanicum* was grown in 250-mL Erlenmeyer flasks with 30 mL Sabouraud culture medium. The inoculum was maintained for 7 days at 30°C, and the mycelial surface was then scraped in presence of 20 mL of saline solution composed by 0.1%[w/v] (NH_4_)_2_SO_4_ + 0.1% [w/v] NH_4_NO_3_ + 0.1% [w/v]MgSO_4_⋅7H_2_O.

### Solid-State Bioprocess (SSB)

The protease were produced by *E. javanicum* under SSB in 250-mL erlenmeyer flasks containing 5 g wheat bran and 9.0 mL saline solution. The medium was sterilized by autoclaving at 121°C for 40 min. One milliliter of the inoculum was added before incubation at 30°C. After 140 h, the bioprocess was stopped, and 40 mL distilled water (4°C) was added to each flask for extracellular enzyme solubilization. This process was aided by maceration with a plastic rod, and the flasks were then agitated in a shaker at 200 rpm for 30 min at 4°C. The material was filtered and centrifuged at 5,000 × *g* for 20 min at 4°C. The supernatant was collected as the enzymatic extract (Hamin Neto et al., 2013).

### Evaluation of Proteolytic Activity with Casein as Substrate

Proteolytic activity was evaluated using casein substrate according to the protocol described by Sarath et al. (1989), with some modifications. One milliliter of 1% (w/v) casein in 50 mM monobasic sodium phosphate buffer (pH 6.5) was combined with100 μL of 50 mM monobasic sodium phosphate buffer (pH 6.5) and 100 μL enzymatic extract. The reaction mixture was incubated for 60 min at 40°C, and 600 μL of 10% (w/v) trichloroacetic acid (TCA) was then added to stop the reaction. The reaction tubes were centrifuged at 10000 × *g* for 10 min at 30°C. The absorbance of the supernatants was then measured relative to the blank controls in cuvettes at 280 nm in a spectrophotometer (GENESYS 10S UV Vis; Thermo Fischer Scientific Inc.). One unit of activity was defined as the amount of the enzyme required to cause an increase of 0.001 of absorbance at 280 nm (Gupta et al., 2002).

### Enzyme Purification by Chromatography

The enzymatic extract was subjected to gel filtration with a column (100 cm × 4 cm) using Sephadex G-50 resin. The equilibration buffer was 50 mM acetate (pH 5.0) with 50 mM NaCl, and the elution flow rate was 0.62 mL/min, regulated by a peristaltic pump (GE-Healthcare). The resin was equilibrated with five column volumes (CV), and 5 mL of sample was then applied. The gradient was isocratic, and 5-mL fractions were collected.

Enzyme fractions were subjected to dialysis with a 14-kDa membrane and 50 mM Tris-HCl buffer (pH 8.0) for 24 h at 4°C. The dialyzed samples (15 mL) were applied to Tricorn columns with 6 mL Resource-Q resin (anionic properties), pre-equilibrated with five CV of 50 mM Tris-HCl buffer (pH 8.0). After application, the resin was washed with the same buffer (two CV), and a linear salt gradient was then started from 0 to 500 mM NaCl using 20 CV of buffer at an elution flow rate of 2 mL/min. One-milliliter fractions were collected. The process was carried out using an ÄKTA Purifier chromatograph (GE-Healthcare) at 25°C.

Proteolytic activity assays and analysis by determination of absorbance at 280 nm were carried out to determine the enzymatic and protein profiles, respectively, and a NanoVue UV/Visible Spectrophotometer (GE-Healthcare) was used to quantify total protein at 280 nm, after each chromatographic process.

### Evaluation of the Purity and Molecular Mass of the Protease

The enzyme fraction purity was evaluated by denaturing polyacrylamide gel electrophoresis (SDS-PAGE) using 12% gels (Laemmli, 1970) stained with silver nitrate (See and Jackowski, 1989). The size of the protease was estimated using Image Lab software version 3.0.

### Determination of the N-Terminal Sequence of the Protease

The N-terminal sequence was determined by cleaving the N-terminal amino acids of proteins and peptides using Edman degradation on a Protein Sequencer PPSQ-33A instrument (Shimadzu Corporation). The PTH-amino acids were separated by high-performance liquid chromatography (HPLC), identified, and analyzed by comparing retention times and UV absorption with a previously quantified standard (Graminho et al., 2013).

### Peptide Substrate Synthesis and Cleavage Site Determination

Fluorescence resonance energy transfer (FRET) peptides were synthesized in an automatic solid-phase peptide synthesizer (Model PSSM-8; Shimadzu Corporation; Hirata et al., 1995) and purified using semi preparative HPLC. Molecular mass determination was carried out using matrix-assisted laser desorption ionization time-of-flight (MALDI-TOF) mass spectrometry with a Microflex LT mass spectrometer (Bruker-Daltonics). Peptide solutions were prepared by resuspending the substrate in dimethyl sulfoxide (DMSO), and their concentrations were determined with a molar extinction coefficient at 365 nm of 17,300 M^-1^ cm^-1^ in a spectrophotometer (Hirata et al., 1995). The scissile bonds of hydrolyzed peptides were identified by the isolation of fragments using analytical HPLC followed by determination of their molecular mass with an LCMS-2020 equipped with an electrospray ionization (ESI) probe (Shimadzu Corporation; Oliveira et al., 2015).

### Biochemical Characterization

Functional biochemical characterization of the purified protease was conducted using FRET peptides. In these peptides, an ortho-aminobenzoic acid (Abz), responsible for the molecular fluorescence emission, was conjugated to the N-terminal amino group, and (2,4-dinitrophenyl)ethylenediamine (EDDnp), a quencher of fluorescence, was conjugated to the C-terminal carboxyl group (Chagas et al., 1991). A fluorescence signal was observed upon cleavage of any peptide bond within the amino acid sequence.

#### Test Conditions for the Enzymatic Reaction during Functional Biochemical Characterization Using FRET Substrates

The enzymatic reaction was carried out using a Lumina fluorescence spectrometer (Thermo Fischer Scientific Inc.) coupled with a Peltier system 4-Position Cell Holder Fluorescence device to control the agitation speed and assay temperature. Reactions with Abz-KLRSSKQ-EDDnp substrate were carried out in a quartz cuvette with an optical path length of 10 mm. The wavelengths were set to λex: 320 nm and λem: 420 nm. Data were collected and analyzed using Luminous software version 3.0.

#### Effects of pH and Temperature

The optimum pH was determined by evaluating different pH values from 4.0 to 10.5 with intervals of 0.5 pH units at 40°C. The buffers used in this analysis were acetate (pH 4.0–5.0), MES (pH 5.5–6.0), HEPES (pH 7.0–8.0), BICINE (pH 8.5–9.0), and CAPS (pH 9.5–10.5). After determining the optimum pH, the optimum temperature was determined from 30 to 75°C, with intervals of 5°C, at pH 6.0. The statistical analysis was performed with one-way ANOVA (analysis of variance) and *post hoc* Tukey, results with *p* < 0.05 were considered significant.

The stability of the enzyme at different pH values was evaluated by incubating the pure enzyme at 25°C for 60 min at various pH values (4.0–10.5), with intervals of 0.5 pH units, followed by reaction at pH 6.0 and 45°C. The buffers used in this analysis were acetate (pH 4.5–5.0), MES (pH 5.5–6.0), HEPES (pH 7.0–8.0), BICINE (pH 8.5–9.0), and CAPS (pH 9.5–10.0).

Thermal stability at different temperatures (30–60°C) and for different incubation times (5, 15, 30, and 60 min) was evaluated by proteases pre-incubation, followed by reaction at pH 6.0 and 45°C.

#### Effects of Ions and Inhibitors

The effects of ions on proteolytic activity were analyzed using NaCl, CoCl_2_, CuCl_2_, CaCl_2_, MgCl_2_, BaCl_2_, and AlCl_3_. The inhibitors tested in this analysis were phenylmethylsulfonyl fluoride (PMSF), ethylenediaminetetraacetic acid (EDTA), iodoacetic acid (IAA), and pepstatin (100 mM stock solutions) according to the protocol described by Dunn (1989). Pure enzyme solution and inhibitors or ions were added to each reaction tube at a final concentration of 10 mM for the inhibitors and ions. The effects of these inhibitors and ions were then analyzed by reaction for 5 min at 45°C, followed by further incubation at pH 6.0 at 45°C. The statistical analysis was performed with one-way ANOVA (analysis of variance) and *post hoc* Tukey, results with *p* < 0.05 were considered significant.

#### Effects of Surfactants

The effects of surfactants on proteolytic activity were analyzed using sodium dodecyl sulfate (SDS), cetyltrimethylammonium bromide (CTAB), TritonX-100, and Tween 20 solutions. Pure enzyme solution and surfactants were added to each reaction at final concentrations of 0.1, 0.2, 0.5, or 1.0% for all surfactants except SDS, which was analyzed at 0.02, 0.04, 0.06, 0.08, and 0.1%. The effects of these surfactants were then analyzed by reaction for 5 min at 45°C, followed by further incubation at pH 6.0 at 45°C.

#### Effects of Urea, Guanidine, and Dithiothreitol (DTT)

Pure enzyme solution and test agents were added to reaction tubes at final concentrations of 10, 25, 50, 100, or 150 mM for urea, guanidine, or DTT. The effects of these three components were then analyzed by reaction for 5 min at 45°C, followed by further incubation at pH 6.0 at 45°C.

### Determination of the Molar Concentration of the Purified Enzyme

The molar concentration of the enzyme was determined by active site titration with the inhibitor phosphoramidon, as described by Klemencic et al. (2000) with modifications. Samples were analyzed in a Lumina fluorescence spectrometer (Thermo Fischer Scientific Inc.) using the substrate Abz-KLRSSKQ-EDDnp at pH 6.0 and 45°C. The wavelengths of fluorescence were adjusted to λex: 320 nm and λem: 420 nm.

### Kinetic Experiments with Synthetic Substrate

Kinetic assays were performed to study the effects of changes in the positions of the amino acids in the substrate and to determine the substrate preference of the anchoring enzyme according to the distribution of amino acids in the peptide sequence. These variations were defined as P_3_, P_2_, P_1_, P′_1_, P′_2_, and P′_3_, represented as follows in enzymes: S_3_, S_2_, S_1_, S′_1_, S′_2_, and S′_3_ (Berger and Schechter, 1970). The kinetic parameters of substrate (Abz-KLXSSKQ-EDDnp) hydrolysis were evaluated with X variations in the indicated positions (P_3_, P_2_, P_1_, P′_1_, P′_2_, and P′_3_).

Enzymatic kinetic data were obtained by addition the substrate to the reaction cuvette with increasing concentrations. The experiments were performed at pH 6.0 and 45°C, and absorbance was measured using a Lumina fluorescence spectrometer (Thermo Scientific), with control of agitation and reaction temperature using a Fluorescence Peltier 4-Position Cell Holder (Thermo Fischer Scientific Inc.). The wavelengths were set to λex: 320 nm and λem: 420 nm.

The kinetic parameters were obtained from the Michaelis-Menten equation calculated by non-linear regression of data from hydrolysis of the substrate using Grafit version 5.0. We analyzed the *K*_M_, *k*_cat_, and *k*_cat_/*K*_M_ to determine the preference of the enzyme for different substrates.

## Results

### Enzyme Purification by Chromatography

**FIGURE 1:**
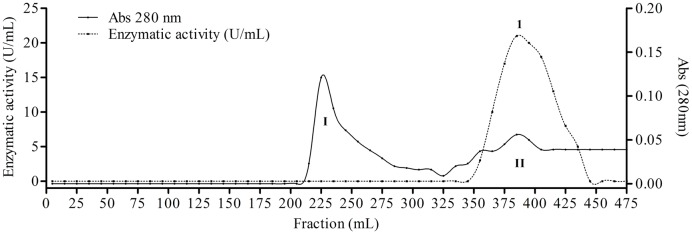
**Elution profile of proteins and protease from *Eupenicillium javanicum* solid-state bioprocess in chromatography of mass exclusion with Sephadex g-50 resin.** The elution buffer was acetate 50 mM, pH 5.0 and 50 mM NaCl. The flow was maintained in 0.62 mL/min and fractions of 5 mL.

**FIGURE 2:**
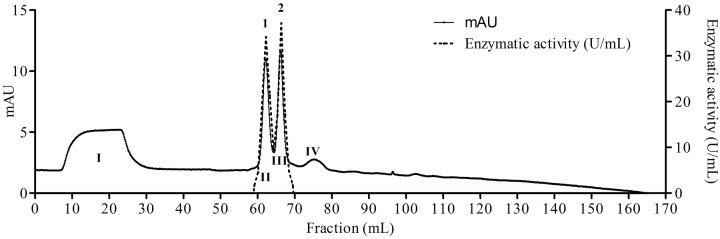
**Elution profile of proteins and protease from *E. javanicum* solid-state bioprocess in chromatography of anion exchange with Resource-Q resin.** The elution buffer was Tris-HCl 50 mM, pH 8.0 and gradient from 0 to 500 mM of NaCl. The flow was maintained in 2 mL/min and fractions of 1 mL.

**FIGURE 3:**
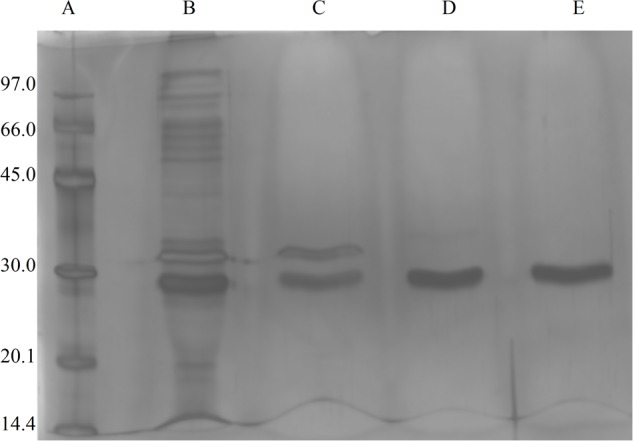
**Image of SDS-PAGE gels (12%) stained with silver nitrate, the sample were from *Eupenicillium javanicum* solid-state bioprocess. (A)** Molecular weight markers, **(B)** crude extract, **(C)** g-50 fraction, **(D)** Resource-Q peak I, and **(E)** Resource-Q peak II.

The fungus *E. javanicum* produced a protease that could be reproducibly purified using the steps described in **Table 1**. A final yield of 27.5% was obtained, with 12.4-fold purification. The elution profile from Sephadex G-50 resin chromatography showed two protein peaks, and peak II (fractions 70–90) exhibited proteolytic activity (**Figure 1**). These fractions were then subjected to anion exchange chromatography (Resource-Q resin), and the elution profile showed four protein peaks. Peaks II (fractions 61–63) and III (fractions 65–67) showed enzymatic activity (**Figure 2**). Peak III was used as the purified enzyme in subsequent steps as this peak showed higher specific activity, fold purification, and yield than peak II. **Figure 3** shows the purity and estimated molecular weight of the enzyme (30 kDa).

**Table 1:** Summary of the purification steps for the *Eupenicillium javanicum* protease, produced by a solid-state bioprocess.

Steps	Total activity (U)	Total protein (mg)	Specific activity (U/mg)	Purification (fold)	Yield (%)
Crude extract	1569.6	16.535	94.9	1.0	100.0
Sephadex G-50	1302.7	2.480	525.3	5.5	83.0
Resource-Q peak 1	394.9	0.448	881.6	9.3	25.2
Resource-Q peak 2	431.9	0.368	1173.7	12.4	27.5

### Determination of the N-Terminal Sequence

**FIGURE 4:**
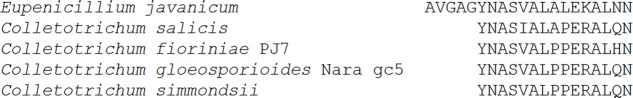
**N-terminal amino acid sequence of the protease isolated from *E. javanicum* solid-state bioprocess and comparisons with other proteases from *Colletotrichum salicis*, *C. fioriniae* PJ7, *C. gloeosporioides* Nara gc5, and *C. simmondsii***.

The N-terminal sequence of the first 20 amino acid residues of the protease from *E. javanicum* was AVGAGYNASVALALEKALNN. The N-terminal sequence was similar (query cover: 75% and identity: 73%) with other proteases secreted from the filamentous fungi *Colletotrichum salicis* (KXH28323.1), *C. fioriniae* PJ7 (XP 007596472.1), *C. gloeosporioides* Nara gc5 (XP 007281997.1), and *C. Simmondsii* (KXH42882.1), as shown in **Figure 4**.

### Biochemical Characterization

#### Effects of pH and Temperature on Enzyme Activity

**FIGURE 5:**
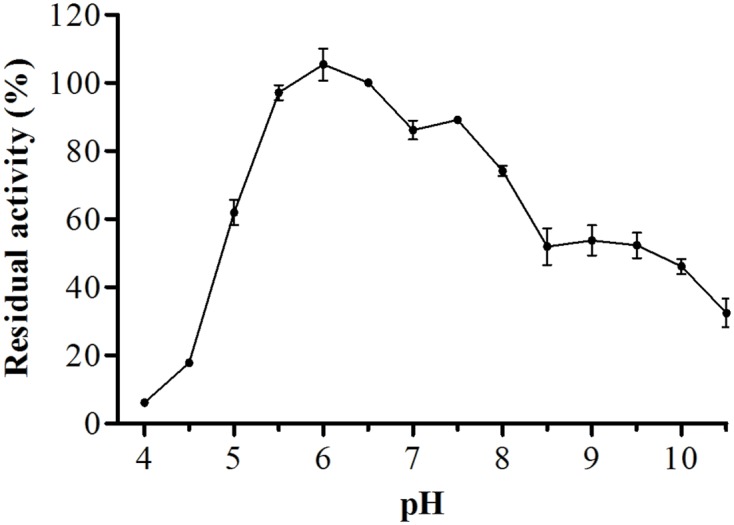
**Optimum proteolytic activity at different pH values (4.0–10.5), the reaction was conducted at 40°C, using Abz-KLRSSKQ-EDDnp as substrate in fluorescence spectrometer**.

**FIGURE 6:**
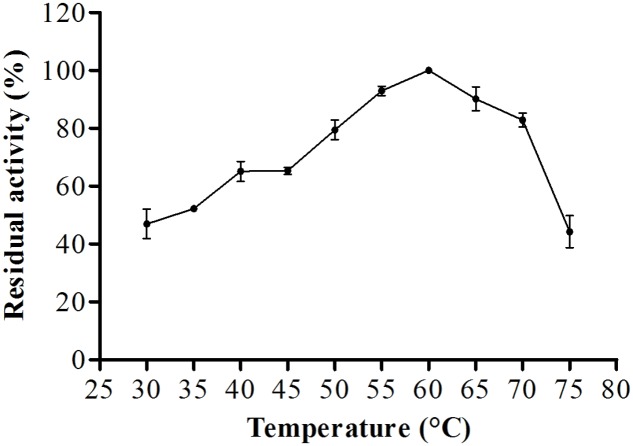
**Optimum proteolytic activity at different temperatures (30–75°C) the reaction was conducted at pH 6.0, using Abz-KLRSSKQ-EDDnp as substrate in fluorescence spectrometer**.

For biochemical characterization of the enzyme, we first analyzed the optimum pH. Interestingly, the protease showed about 75% or more residual activity from pH 5.5–8.0, with 97% or more between pH 5.5 and 6.5, the optima pH was at 6.0, with The optimum temperature was 60°C, with statistical difference compared with all pH values except at 6.5, *p* < 0.05 (**Figure 5**). Proteolytic activity was lower at more extreme pH values. Enzymatic assays with incubation at different temperatures showed an increase from 30 to 60°C, followed by a drop in activity at temperatures over 60°C. The enzyme maintained and 80% or more activity from 50 to 70°C. The optimum temperature was 60°C, with statistical difference compared with all temperatures tested, *p* < 0.05 (**Figure 6**).

**FIGURE 7:**
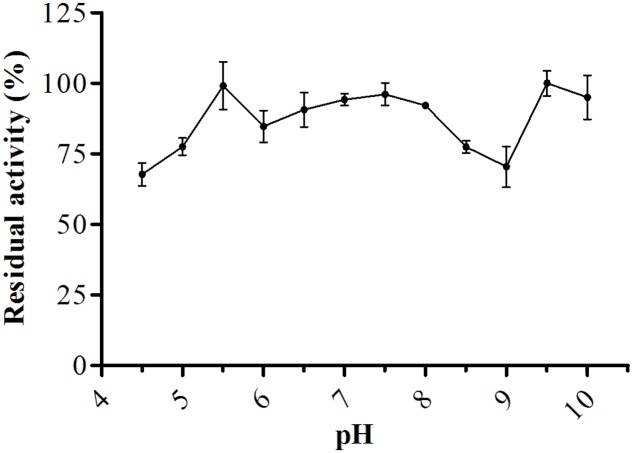
**Protease stability after pre-incubation at different pH values (4.5–10.0) after 60 min at 25°C, the reaction was conducted at pH 6.0 and 45°C, using Abz-KLRSSKQ-EDDnp as substrate in fluorescence spectrometer**.

**FIGURE 8:**
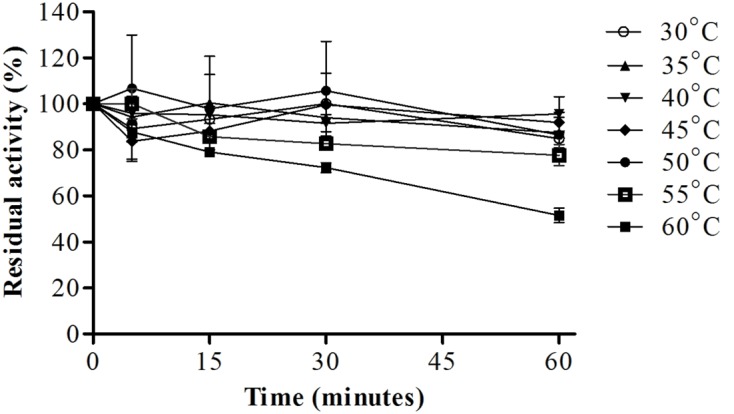
**Protease stability after pre-incubation at different temperatures (30–60°C) for different times (5, 15, 30, and 60 min), the reaction was conducted at pH 6.0 and 45°C, using Abz-KLRSSKQ-EDDnp as substrate in fluorescence spectrometer**.

Analysis of protease stability at different pH values showed that the enzyme had greater stability from pH 4.5–10.0 with maintenance of about 67% or more of it stability after 60 min of incubation at 25°C (**Figure 7**). The protease showed great thermal stability during a 60-min incubation; from 30 to 55°C, the residual activity was about 80% or more, and at 60°C, the residual activity of the enzyme was 50% (**Figure 8**). Thus, these results suggested that the enzyme was thermostable despite being produced by a mesophilic fungus.

#### Effects of Ions and Inhibitors

Metal ions can either positively and negatively affect protease activity. In this study, we found that 10 mM sodium, barium, or cobalt increased proteolytic activity by 52, 24, and 24%, only sodium showed statistical difference compared with control, *p* < 0.0001 (**Table 2**), respectively, while aluminum decreased the original proteolytic activity by 16%. Additionally, EDTA inhibited the proteolytic activity by about 69% with statistical difference, *p* < 0.0001, whereas PMSF, IAA, and pepstatin inhibited proteolytic activity by 30, 27, and 20%, respectively, (**Table 3**). These results suggested that the enzyme maybe a metalloprotease.

**Table 2:** Effects of 10 mM of different ions on the hydrolysis of Fluorescence resonance energy transfer (FRET) substrate by the purified *E. javanicum* protease.

Salts	Residual proteolytic activity (%)
Control	100 ± 12.74
NaCl	152 ± 10.94
BaCl_2_	124 ± 12.21
CoCl_2_	124 ± 3.92
CuCl_2_	98 ± 3.43
MgCl_2_	96 ± 2.13
CaCl_2_	94 ± 6.32
AlCl_3_	84 ± 12.39

*The values were presented as media ± standard deviation*.

*The reaction was pre incubated for 5 min at 45°C, followed by reaction at pH 6.0 at 45°C with Abz-KLRSSKQ-EDDnp substrate*.

**Table 3:** Effects of 10 mM different inhibitors on the hydrolysis of FRET substrate by the purified *E. javanicum* protease.

Inhibitors	Residual proteolytic activity (%)
Control	100 ± 4.80
EDTA	31 ± 0.84
PMSF	70 ± 1.55
Iodoacetic acid	73 ± 5.86
Pepstatin	80 ± 4.89

*The values were presented as media ± standard deviation*.

*The reaction was pre incubated for 5 min at 45°C, followed by reaction at pH 6.0 at 45°C with Abz-KLRSSKQ-EDDnp substrate*.

#### Effects of Surfactants

**FIGURE 9:**
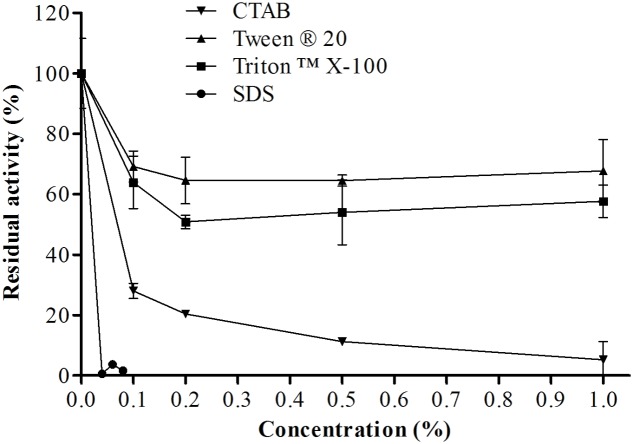
**Effects of different concentrations of a variety of surfactants (CTAB, SDS, Triton X-100, and Tween 20) on the proteolytic activity of *E. javanicum* protease, the reaction was conducted at pH 6.0 and 45°C, using Abz-KLRSSKQ-EDDnp as substrate in fluorescence spectrometer**.

**Figure 9** shows the residual activity of the protease isolated from *E. javanicum* in the presence of surfactants. The enzyme was stable at different concentrations of Triton X-100 and Tween 20; at the maximum concentration tested (1%), the protease maintained about 68 and 58% of its activity, respectively. Increases in CTAB and SDS concentrations decreased protease activity; specifically, the protease retained about 28% activity in the presence of 0.1% CTAB and about 30% activity in the presence of 0.02% SDS.

#### Effects of Urea, Guanidine, and DTT

**FIGURE 10:**
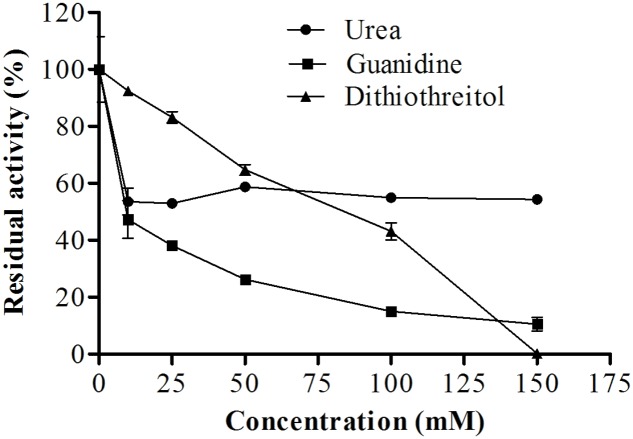
**Effects of different concentrations of dithiothreitol, guanidine, and urea on the proteolytic activity of *E. javanicum* protease, the reaction was conducted at pH 6.0 and 45°C, using Abz-KLRSSKQ-EDDnp as substrate in fluorescence spectrometer**.

As shown in **Figure 10**, urea, guanidine, and DTT also affected proteolytic activity. Increased concentrations of DTT resulted in decreased enzymatic activity, maintaining about 40% of enzyme activity when the concentration of DTT was 100 mM. In the presence of 150 mM urea, the enzyme maintained about 54% of its original activity. Interestingly, guanidine had a greater effect, reducing the activity of the enzyme by only 10% when used at a concentration of 150 mM.

### Kinetic Experiments with a Synthetic Substrate

**Table 4** shows the catalytic specificity of the protease for S_1_, S_2_, and S_3_ subsites in relation to replacement of amino acids at P_1_, P_2_, and P_3_ positions using a FRET peptide series based on the sequence Abz-KLRSSKQ-EDDnp (considering the cleavage between Arg and Ser). The position P_1_ provided higher catalytic efficiency with arginine, phenylalanine, and methionine (2,146, 2,043, and 1,843 mM^-1^⋅seg^-1^, respectively). Moreover, the protease exhibited higher affinity when glutamine was in this amino acid position, with a *K*_M_ of 0.004 mM. Most of the substrates were cleaved between P′_1_ and P′_2_; in the presence of arginine and glutamine, the substrate was cleaved as follows: P_1_↓P′_1_↓P′_2_. The best values for the S_2_ subsite were with substrates containing valine and arginine at the P_2_ substrate position; the catalytic efficiencies were 2,141 and 1,850 mM^-1^⋅seg^-1^. The enzyme showed high affinity for phenylalanine, with a *K*_M_ of 0.012 mM. The substrates were cleaved between P′_1_ and P′_2_, except for phenylalanine and tyrosine, which were cleaved as follows: P_1_↓P′_1_↓P′_2_. The kinetic parameters of the protease at S_3_ showed that the presence of hydrophobic amino acids, such as valine, isoleucine, and alanine, led to the highest catalytic efficiencies (4,539, 3,886, and 2,836 mM^-1^⋅seg^-1^, respectively). The enzyme showed high affinity for valine, with a *K*_M_ of 0.010 mM. All substrates were cleaved at two positions, P_1_↓P′_1_↓P′_2_, except in the presence of serine, where the cleavage pattern was P′_1_↓P′_2_; that of arginine was not determined.

**Table 4:** Kinetic parameters for the hydrolysis by the *E. javanicum* protease of the peptide series derived from reference peptide Abz-KLRSSKQ-EDDnp modified at P_1_, P_2_, and P_3_ positions.

	*k*_cat_ (s^-1^)	*K*_M_ (mM)	*k*_cat_/*K*_M_ (mM^-1^ s^-1^)
P_1_ position			
Abz-KL**R**↓45%S↓55%SKQ-EDDnp	27.9 ± 0.2	0.013 ± 0.001	2,146 ± 167
Abz-KL**F**SSKQ-EDDnp	42.9 ± 4.8	0.021 ± 0.005	2,043 ± 251
Abz-KL**M**S↓SKQ-EDDnp	12.9 ± 0.4	0.007 ± 0.001	1,843 ± 242
Abz-KL**Y**S↓SKQ-EDDnp	4.5 ± 0.5	0.010 ± 0.003	450 ± 89
Abz-KL**H**S↓SKQ-EDDnp	3.3 ± 0.2	0.007 ± 0.000	471 ± 24
Abz-KL**A**↓SSKQ-EDDnp	6.9 ± 2.2	0.019 ± 0.013	363 ± 178
Abz-KL**Q**↓S↓SKQ-EDDnp	1.5 ± 0.0	0.004 ± 0.001	375 ± 51
Abz-KL**V**S↓SKQ-EDDnp	4.5 ± 0.3	0.021 ± 0.005	214 ± 42
P_2_ position			
Abz-KL**R**↓45%S↓55%SKQ-EDDnp	27.9 ± 0.2	0.013 ± 0.001	2,146 ± 167
Abz-K**V**RS↓SKQ-EDDnp	36.4 ± 0.3	0.017 ± 0.001	2,141 ± 93
Abz-K**R**RS↓SKQ-EDDnp	40.7 ± 1.1	0.022 ± 0.001	1,850 ± 59
Abz-K**I**R↓50%S↓50%SKQ-EDDnp	49.7 ± 3.2	0.029 ± 0.003	1,714 ± 86
Abz-K**M**RS↓SKQ-EDDnp	35.6 ± 0.5	0.021 ± 0.001	1,695 ± 37
Abz-K**Y**RS↓50%S↓50%KQ-EDDnp	42.7 ± 3.2	0.026 ± 0.004	1,642 ± 350
Abz-K**K**RS↓SKQ-EDDnp	59.3 ± 12.1	0.039 ± 0.008	1,520 ± 6
Abz-K**A**RS↓SKQ-EDDnp	28.1 ± 1.5	0.032 ± 0.003	878 ± 29
Abz-K**F**R↓SSKQ-EDDnp	8.1 ± 0.7	0.012 ± 0.004	675 ± 193
Abz-K**P**RS↓SKQ-EDDnp	19.8 ± 5.4	0.033 ± 0.015	600 ± 114
Abz-K**S**RS↓SKQ-EDDnp	10.7 ± 1.4	0.031 ± 0.009	345 ± 63
Abz-K**E**RS↓SKQ-EDDnp	4.3 ± 1.0	0.020 ± 0.005	215 ± 6
P_3_ position			
Abz-KL**R**↓45%S↓55%SKQ-EDDnp	27.9 ± 0.2	0.013 ± 0.001	2,146 ± 167
Abz-**V**LR↓73%S↓27%SKQ-EDDnp	45.39 ± 0.66	0.010 ± 0.000	4,539 ± 28
Abz-**I**LR↓71%S↓29%SKQ-EDDnp	42.75 ± 4.12	0.011 ± 0.002	3,886 ± 353
Abz-**A**LR↓S↓SKQ-EDDnp	36.87 ± 1.69	0.013 ± 0.001	2,836 ± 133
Abz-**R**LRSSKQ-EDDnp	19.17 ± 1.97	0.013 ± 0.001	1,475 ± 227
Abz-**H**LR↓41%S↓59%SKQ-EDDnp	27.11 ± 0.18	0.021 ± 0.001	1,290 ± 63
Abz-**G**LR↓41%S↓59%SKQ-EDDnp	18.55 ± 0.67	0.015 ± 0.001	1,237 ± 53
Abz-**Q**LR↓33%S↓67%SKQ-EDDnp	20.59 ± 0.36	0.019 ± 0.001	1,084 ± 65
Abz-**Y**LR↓39%S↓61%SKQ-EDDnp	12.99 ± 1.26	0.014 ± 0.002	928 ± 220
Abz-**F**LR↓44%S↓56%SKQ-EDDnp	14.21 ± 0.43	0.018 ± 0.004	789 ± 162
Abz-**S**LRS↓SKQ-EDDnp	10.32 ± 0.49	0.018 ± 0.001	573 ± 19
Abz-**W**LR↓40%S↓60%SKQ-EDDnp	4.38 ± 1.74	0.013 ± 0.008	337 ± 68

*The values were presented as media ± standard deviation. The letter marked in bold refers the amino acids that were exchanged in each substrate*.

**Table 5** shows values of the protease kinetic parameters at S′_1_, S′_2_, and S′_3_ subsites related to substrate substitution at P′_1_, P′_2_, and P′_3_ positions. Analysis of the S′_1_ data showed that the substrate containing tyrosine was the most hydrolyzed, with a catalytic efficiency of 87,849 mM^-1^⋅seg^-1^, followed by arginine (catalytic efficiency: 18,914 mM^-1^⋅seg^-1^) and lysine (14,876 mM^-1^⋅seg^-1^). All substrates evaluated could be hydrolyzed by protease, particularly tyrosine, which resulted in the highest enzyme affinity (*K*_M_: 0.007 mM). The most frequently cleaved site was P′_1_↓P′_2_, except in the presence of valine (P_2_↓P_1_), lysine (P′_1_↓P′_2_↓P′_3_), and Arg or Met (P_2_↓P_1_P′_1_↓P′_2_). The protease kinetic parameters in isoleucine presence at S′_2_ subsite provided high catalytic efficiency with 13,700 mM^-1^⋅seg^-1^. The affinity of the enzyme was higher in the presence of isoleucine and tyrosine, *K*_M_ 0.003. Additionally, the most frequently cleaved sites were P_2_↓P1_↓_P′_1_ for proline and tyrosine; P_1_↓P′_1_↓P′_2_ for alanine, arginine, glutamine, glutamic acid, and histidine; P_2_↓P_1_↓P′_1_↓P′_2_ for glycine; and P_2_↓P_1_↓P′_1_↓P′_2_↓P′_3_ for histidine. The S′_3_ subsite had higher catalytic efficiency in the presence of tyrosine and phenylalanine (3,833 and 2,085 mM^-1^⋅seg^-1^, respectively). The cleavage was different for all substrates.

**Table 5:** Kinetic parameters for the hydrolysis by the *E. javanicum* protease of the peptide series derived from reference peptide Abz-KLRSSKQ-EDDnp modified at P′_1_, P′_2_, and P′_3_ positions.

	*k*_cat_(s^-1^)	*K*_M_ (mM)	*k*_cat_/*K*_M_ (mM^-1^ s^-1^)
P′_1_ position			
Abz-KL**R**↓45%S↓55%SKQ-EDDnp	27.9 ± 0.2	0.013 ± 0.001	2,146 ± 167
Abz-KLR**Y**↓SKQ-EDDnp	614.94 ± 24.36	0.007 ± 0.001	87,849 ± 15571
Abz-KL↓49%R**R**↓51%SKQ-EDDnp	132.40 ± 1.88	0.007 ± 0.001	18,914 ± 3736
Abz-KLR**K**↓46%S↓54%KQ-EDDnp	119.01 ± 4.05	0.008 ± 0.001	14,876 ± 1584
Abz-KLR**F**↓SKQ-EDDnp	71.95 ± 6.04	0.008 ± 0.001	8,994 ± 891
Abz-KLR**H**↓SKQ-EDDnp	116.95 ± 2.23	0.016 ± 0.002	7,309 ± 664
Abz-KLR**A**↓SKQ-EDDnp	141.87 ± 19.61	0.022 ± 0.004	6,449 ± 146
Abz-KL↓R**V**SKQ-EDDnp	63.32 ± 3.46	0.023 ± 0.001	2,753 ± 22
Abz-KL↓46%R**M**↓54%SKQ-EDDnp	12.92 ± 0.38	0.007 ± 0.001	1,846 ± 242
Abz-KLR**E**↓SKQ-EDDnp	44.79 ± 0.32	0.027 ± 0.002	1,659 ± 134
Abz-KLR**P**↓SKQ-EDDnp	26.27 ± 2.36	0.019 ± 0.002	1,383 ± 57
Abz-KLR**Q**↓SKQ-EDDnp	14.30 ± 0.59	0.011 ± 0.002	1,300 ± 212
Abz-KLR**G**↓SKQ-EDDnp	5.71 ± 0.51	0.017 ± 0.000	336 ± 20
P′_2_ position			
Abz-KL**R**↓45%S↓55%SKQ-EDDnp	27.9 ± 0.2	0.013 ± 0.001	2,146 ± 167
Abz-KLR↓S**I**KQ-EDDnp	41.1 ± 3.6	0.003 ± 0.001	13,700 ± 2538
Abz-KLR↓49%S↓51%**R**KQ-EDDnp	52.2 ± 0.3	0.005 ± 0.000	10,440 ± 139
Abz-KLR↓S**Y**KQ-EDDnp	24.1 ± 2.7	0.003 ± 0.000	8,033 ± 562
Abz-KLR↓S**M**KQ-EDDnp	60.6 ± 4.0	0.007 ± 0.001	8,657 ± 370
Abz-KLR↓21%S↓79%**A**KQ-EDDnp	54.9 ± 2.2	0.012 ± 0.001	4,575 ± 250
Abz-KLR↓S**V**KQ-EDDnp	41.1 ± 2.1	0.010 ± 0.001	4,110 ± 139
Abz-KLR↓38%S↓62%**Q**KQ-EDDnp	41.2 ± 1.7	0.011 ± 0.001	3,745 ± 153
Abz-KLR↓S**K**KQ-EDDnp	27.7 ± 2.4	0.011 ± 0.002	2,518 ± 154
Abz-KL↓R↓S↓**H**↓KQ-EDDnp	17.7 ± 2.4	0.009 ± 0.001	1,967 ± 135
Abz-KL↓45%R↓55%S**W**KQ-EDDnp	14.8 ± 1.0	0.013 ± 0.002	1,138 ± 65
Abz-KLR↓58%S↓42%**E**KQ-EDDnp	8.6 ± 1.5	0.010 ± 0.004	860 ± 228
Abz-KL↓46%R↓54%S**F**KQ-EDDnp	15.5 ± 2.4	0.018 ± 0.004	861 ± 55
Abz-KL↓R↓S↓**G**KQ-EDDnp	11.2 ± 2.2	0.022 ± 0.006	509 ± 36
Abz-KLR↓S**P**KQ-EDDnp	5.0 ± 0.6	0.026 ± 0.005	192 ± 13
P′_3_ position			
Abz-KL**R**↓45%S↓55%SKQ-EDDnp	27.9 ± 0.2	0.013 ± 0.001	2,146 ± 167
Abz-KL↓27%R↓20%S↓53%S**Y**Q-EDDnp	23.0 ± 0.6	0.006 ± 0.000	3,833 ± 101
Abz-KLR↓29%S↓71%S**F**Q-EDDnp	27.1 ± 1.2	0.013 ± 0.003	2,085 ± 451
Abz-KLRS↓S**I**Q-EDDnp	13.0 ± 3.1	0.008 ± 0.000	1,625 ± 290
Abz-KL↓32%R↓18%S↓50%S**V**Q-EDDnp	17.4 ± 2.4	0.019 ± 0.003	916 ± 12
Abz-KLR↓SS**W**Q-EDDnp	9.8 ± 0.0	0.014 ± 0.000	700 ± 12
Abz-KL↓81%R↓19%SS**A**Q-EDDnp	9.6 ± 0.9	0.026 ± 0.003	369 ± 6
Abz-KL↓R↓S↓S**S**Q-EDDnp	4.2 ± 0.6	0.018 ± 0.007	233 ± 72

*The values were presented as media ± standard deviation. The letter marked in bold refers the amino acids that were exchanged in each substrate*.

## Discussion

In this study, we evaluated the optimum pH and temperature, stability, and kinetic parameters of a protease isolated from solid-state fermentation of *E. javanicum*. According to the literature, the *Penicillium* genus has the potential to produce different proteases; for example, *Penicillium waksmanii* (Graminho et al., 2013), *P. digitatum* (Aissaoui et al., 2014), and *P. italicum* (Abidi et al., 2014) produce serine proteases under submerged bioprocesses, whereas *P. waksmanii* and *P. corylophilum* (Da Silva et al., 2013) also produce serine proteases under solid-state fermentation. Moreover, similar to *E. javanicum*, *Penicillium* ssp. produces metalloproteases during solid-state fermentation (El-Gendy, 2010).

### Enzyme Purification by Chromatography

Purification processes can vary depending on the specific enzyme being isolated. For example, the different chromatography steps can be modified according to the stability of the enzyme in response to process conditions, influencing the recovery and purification fold, that influence directly in cost of industrial use. Previous studies have described appropriate purification parameters for enzymes from *P. italicum* (Abidi et al., 2014), *Beauveria* sp. (Shankar et al., 2011), and *Trichoderma harzianum* (Savitha et al., 2011). Additionally, the protease purified from submerged medium of *Aspergillus oryzae* KSK-3 had 182.7-fold purification, but a recovery of only 0.005% (Shirasaka et al., 2012). A metalloprotease isolated from *Penicillium* spp. showed lower recovery and purification than that from *E. javanicum* (4.93 and 3.56%, respectively; El-Gendy, 2010). Therefore the purification of the metalloprotease from *E. javanicum* presented results better than some protease purifications.

Previous studies have shown that other metalloproteases exhibit variable molecular weights. For example, Bersanetti et al. (2005) isolated a 51.5-kDa metalloprotease from *Serratia proteamaculans* culture medium, and a 19-kDa metalloprotease was produced by *Penicillium* spp. under solid-state fermentation (El-Gendy, 2010). Additionally, *Termitomyces clypeatus* (Majumder et al., 2015) and *Bacillus* sp TKU004 (Wang et al., 2006) produce metalloproteases with molecular masses similar to those of the enzyme secreted by *E. javanicum* in our study (30 kDa).

### Biochemical Characterization

#### Effects of pH and Temperature on Enzyme Activity

Proteases have several properties that may facilitate their use in industrial applications, including optimal temperature, optimal pH, specificity, activity, and stability (Mahajan and Badgujar, 2010). Similar to *E. javanicum*, many microorganisms are able to produce neutral proteases. For example, *P. digitatum* (Aissaoui et al., 2014), *P. italicum* (Abidi et al., 2014), *Graphium putredinis*, and *Trichoderma harzianum* (Savitha et al., 2011) produce serine proteases with an optimum pH at 7.0. Additionally, the metalloproteases from *Microbacterium* sp. (Thys and Brandelli, 2006), *Penicillium* spp. (El-Gendy, 2010), *Thermoascus aurantiacus* (Merheb-Dini et al., 2009), and *Streptomyces septatus* TH-2 (Hatanaka et al., 2005) produce enzymes with a neutral pH optimum.

Additionally, microorganisms are able to secrete enzymes that can function in wide temperature range. As previously reported, *P. italicum* (Abidi et al., 2014), *P. chrysogenum* (Chen et al., 2013), *Botrytis cinerea* (Abidi et al., 2011), *Graphium putredinis*, and *Trichoderma harzianum* (Savitha et al., 2011) produce proteases with an optimum temperature of 50°C, whereas the serine protease from *P. waksmanii* (Graminho et al., 2013) exhibits maximum activity at 35°C. A metalloprotease isolated from *Thermoascus aurantiacus* was found to have high proteolytic activity at 75°C (Merheb-Dini et al., 2009), and that from *Termitomyces clypeatus* was found to have high proteolytic activity at 45°C (Majumder et al., 2015).

The stability of enzymes at different pH values and temperatures also affects the applicability of the enzyme in industrial processes. Enzymes that are stable at a wide range of temperatures and pH values may have broad industrial applications. For example, proteases produced by *P. italicum* (Abidi et al., 2014) and *Beauveria* sp. (Shankar et al., 2011) exhibit high activity from pH 4.0–11.0 and 3.0–11.0, respectively. In contrast, the protease produced by *Botrytis cinerea* is stable from pH 6.0–9.0 (Abidi et al., 2011). The metalloproteases from *Penicillium* spp. show stability in a lower pH range (6.0–8.0 and 6.0–11.0; El-Gendy, 2010). In our study, we found that the enzyme from *E. javanicum* was stable at a wide pH range. Moreover, the thermal stability of our enzyme was similar to that of proteases from *P. italicum*, which maintained about 80% activity at 40 and 50°C and 60% activity at 60°C after 60 min (Abidi et al., 2014). In contrast, the proteases from *Boritrys cinerea* maintained only 10% activity at 60°C after 60 min (Abidi et al., 2011), whereas the proteases from *Graphium putredinis* and *Trichoderma harzianum* were stable for only 15 min at 60°C (Savitha et al., 2011). A metalloprotease produced by *Microbacterium* sp showed similar behavior to that isolated from *E. javanicum*, maintaining more than 80% activity at 55°C and about 70% activity at 60°C after 60 min (Thys and Brandelli, 2006). Thus, the enzyme produced by *E. javanicum* was more stable than some proteases from thermophilic fungi. This resistance to high temperatures is interesting for industrial applications of the enzyme.

Some food processes use high temperatures to avoid the contamination with microorganisms. Proteases that act in high temperatures can be applied in meat tenderization, clean up of DNA before PCR (Bruins et al., 2001) and textile industry to remove gum (Srilakshmi et al., 2014).

#### Effects of Ions and Inhibitors

Some proteases, particularly metalloproteases, may be influenced by the presence of metal ions. Therefore, researchers have evaluated the effects of ions on proteolytic activity. For example, the metalloprotease produced under solid-state fermentation by *Bacillus* sp. exhibits improved activity in the presence of sodium (Saxena and Singh, 2011). Additionally, proteases from *P. waksmanii* (Graminho et al., 2013) and *Botrytis cinerea* (Abidi et al., 2011) show increased activity in the presence of barium. We observed the same tendencies in the metalloprotease isolated in this study. Similarly, a protease from *Beauveria* sp. exhibits increased activity after pre-incubation with cobalt (Shankar et al., 2011). In contrast, the presence of aluminum decreases the activity of proteases from *P. waksmanii* (Graminho et al., 2013) and *Aspergillus oryzae* KSK-3 (Shirasaka et al., 2012). Finally, inhibitors, such as EDTA, can be used to classify the protease according to it catalytic site. EDTA is a metal chelating agent, and proteolytic activity of a metalloprotease is linked to presence of divalent metal ions, which can be directly related to the catalytic site or structure. In our study, EDTA inhibited the activity of our enzyme, whereas barium enhanced the activity of our enzyme, suggesting that the protease isolated from *E. javanicum* in this study was a metalloprotease.

#### Effect of Surfactants

In order to utilize proteases in the detergent industry, the enzymes must be highly active and stable at a high pH and temperature and must show compatibility with other chelating and oxidant agents added to the detergent (Rani et al., 2013). Detergents and surfactants may disrupt hydrophobic interactions within the protein structure and negatively or positively affect proteolytic activity. Enzymes produced by *P. digitatum* maintain about 64% activity in the presence of Tween (Aissaoui et al., 2014). In contrast, enzymes produced by *Myceliophthora* sp. show increased activity in the presence of Tween (117% of residual activity; Zanphorlin et al., 2011). In addition, the protease produced by *P. waksmanii* exhibits only 43% activity in the presence of this detergent (Graminho et al., 2013). The protease from *P. digitatum* exhibits about 70% residual activity in the presence of Triton (Aissaoui et al., 2014), whereas that from *Thermoascus aurantiacus* has no stability in the presence of the same surfactant (Merheb-Dini et al., 2009). Proteases produced by *P. waksmanii* have been shown to retain about 43% activity in the presence of 0.8% Triton (Graminho et al., 2013). Some proteases produced by *P. waksmanii* (Graminho et al., 2013) and *Myceliophthora* sp (Zanphorlin et al., 2011) have no activity when SDS is added to the solution, whereas the protease produced by *Botrytis cinerea* (Abidi et al., 2011) shows increased stability in the presence of 1% SDS.

#### Effects of Urea, Guanidine, and DTT

Reducing agents such as DTT may break disulfide bonds from sulfhydryl groups. The tertiary protein structure depends on these connections and hydrophobic interactions to stabilize the protein (Vieille and Zeikus, 2001). Indeed, disulfide bonds are important for the structure and proteolytic activity of the metalloprotease produced by *E. javanicum*. Additionally, the enzyme from *Thermoascus aurantiacus* also showed a decrease in activity as the concentration of DTT increased (Merheb-Dini et al., 2009). Additionally, the enzyme produced by *P. waksmanii* maintained only 15% activity in the presence of 20 mM DTT (Graminho et al., 2013); thus, this enzyme was less stable than that produced by *E. javanicum*. Hydrogen bonds also help to maintain the high order structure of proteins. Chaotropic agents, such as urea and guanidine, can disrupt these connections and induce changes in protein conformation (Adrio and Demain, 2014), thereby affecting proteolytic activity, as was observed for the metalloprotease secreted by *E. javanicum*.

### Kinetic Experiments with a Synthetic Substrate

In this study, we performed kinetic analysis to determine the specificity of the protease to certain substrates, which can be targets in industrial applications. Replacement of some amino acids at the P_1_ and P_2_ positions of the Abz-KLRSSKQ-EDDnp substrate blocked substrate cleavage, suggesting that the enzyme required specific amino acids at this position. Analysis of the amino acid residues at P_3_ position and the catalytic efficiency of the enzyme showed that there was a preference for non-polar amino acids. In contrast, all the amino acid residues allowed substrate cleavage at the P_3_ position; thus, there was lower enzymatic specificity at P_3_ compared with those at P_1_ and P_2_.

Replacement of amino acids at P′_1_ showed that polar amino acids positively influenced catalytic efficiency, with tyrosine having the greatest effects. At P′_2_, the subsite alternated between polar and non-polar substrates; therefore, this position was non-specific for the enzyme. Additionally, analysis of the kinetics at the P′_3_ position showed that there was a preference for amino acids with an aromatic ring. Some amino acids also blocked cleavage completely, demonstrating that this position required specific amino acids. For positions at the “primed” side, the enzyme showed greater catalytic efficiency than when there were changes at the “unprimed” side positions. There were no similarities among the P_1_, P_2_, and P_3_ positions. Additionally, analysis of the specificity of proteases using FRET substrates showed that enzymes secreted by *P. waksmanii* (Graminho et al., 2013) and *Myceliophthora* sp. (Zanphorlin et al., 2011) showed the highest catalytic efficiency at the “unprimed” side, with Ile at P_1_ and Ile at P_2_, respectively. In contrast, proteases from *Myceliophthora thermophila* (Hamin Neto et al., 2015) and *Aspergillus fumigatus* (Da Silva et al., 2014) showed the highest catalytic efficiency at the “primed” side, with Ala at P′_2_ and Leu at P′_3_, respectively. Furthermore, different enzymes show specificity to the various types of substrates, highlighting the importance of kinetic assays.

In summary, the metalloprotease isolated from *E. javanicum* during solid-state fermentation exhibited characteristics that were important and desirable for a variety of industrial applications. These findings may provide important insights into the availability of novel enzyme resources for industrial processes; it is necessary studies in each industrial area to suggest an application.

## Author Contributions

Bioprocess, purification, biochemical characterization and kinetic experiment (YH and HC). Peptide substrate synthesis and cleavage site determination (LdO, JdO, MJ, and LJ). Determination the N-terminal sequence (EA).

## Conflict of Interest Statement

The authors declare that the research was conducted in the absence of any commercial or financial relationships that could be construed as a potential conflict of interest.
